# Artificial Intelligence and Machine Learning for Identifying Social Determinants of Health in Low‐Income Populations Within United States Health Systems: A Scoping Review

**DOI:** 10.1002/hsr2.73083

**Published:** 2026-08-26

**Authors:** Anthony Vincent Razzano

**Affiliations:** ^1^ Department of Public Health, Zuckerberg College of Health Sciences University of Massachusetts Lowell Lowell MA USA

**Keywords:** artificial intelligence, electronic health records, health equity, low‐income populations, machine learning, natural language processing, predictive modeling, scoping review, social determinants of health

## Abstract

**Background and Aims:**

Low‐income populations in the United States experience disproportionate exposure to adverse social conditions. This scoping review examined artificial intelligence (AI) and machine learning (ML) approaches used in United States health systems to identify financial hardship, housing instability, food insecurity, neighborhood deprivation, and other social determinants of health (SDOH), and characterized interventions implemented in response.

**Methods:**

Following the Arksey and O'Malley framework and PRISMA‐ScR guidance, MEDLINE, CINAHL Plus with Full Text, PsycINFO, and Academic Search Ultimate were searched for peer‐reviewed studies published from January 2010 through January 2026. The primary reviewer screened 209 unique records, and two additional reviewers reviewed screening decisions. Disagreements were resolved through discussion and majority vote. Seventeen studies met the inclusion criteria.

**Results:**

Studies were conducted in academic medical centers, integrated delivery networks, safety‐net hospitals, Veterans Health Administration facilities, specialty clinics, and Medicaid administrative environments. Methods included predictive ML modeling, natural language processing, and unsupervised clustering, with electronic health records used in 16 of 17 studies. Only one study evaluated a structured intervention: a proactive financial assistance program for ambulatory oncology patients at risk of financial hardship and unmet social needs. The remaining studies focused primarily on risk identification or model development. External validation was uncommon, and several studies reported differential performance across socioeconomic or racial and ethnic groups.

**Conclusion:**

AI and ML may support SDOH risk identification in low‐income populations, but evidence of generalizability, fairness, and translation into effective interventions remains limited. Future research should prioritize external validation, equity‐focused evaluation, and rigorous testing of interventions triggered by identified social risks.

## 1 Introduction

Health disparities among low‐income populations in the United States reflect persistent structural failures in the modern healthcare system. Social determinants of health (SDOH) encompass non‐medical conditions including birth, growth, work, and living environment, and are primary drivers of health outcomes for economically disadvantaged groups in clinical settings [1]. Global data sectors increasingly shape public health strategy, and scholars apply a data equity lens to address disparities. Degraded public health data infrastructure in the United States makes such efforts urgent [1].

AI and ML technologies identify and quantify SDOH‐driven risks. Big data systems influence population health outcomes by reshaping how providers understand health determinants and structure interventions [2]. Medical AI publications grew rapidly after 2017, with research output tracking national economic and innovation capacity even as high‐income and low‐income nations retain unequal access to these tools [3]. Uneven distribution of technological capability mirrors existing health inequities and creates a central tension for this review.

Electronic health records (EHRs) provide the foundational data infrastructure for AI‐driven SDOH identification. Expanded computing capacity allows AI algorithms to analyze large patient populations [4]. ML applied to linked EHR and census data outperforms traditional statistical methods in detecting complex and longitudinal social determinants. One ML analysis of 90,000 dental records identified significant associations between social determinants and periodontal disease that conventional methods frequently miss [5]. Deep learning applied to national health surveys reveals inverse relationships between income, education, and chronic disease, findings directly relevant to low‐income populations in safety‐net health systems with elevated rates of hypertension and diabetes [6].

AI and big data methodologies extend well beyond EHR analysis into clinical domains directly relevant to low‐income health. Integrating big data analytics with genomic sequencing and SDOH data improves personalized cancer care and patient safety [7]. Large health systems apply predictive analytics to identify high‐risk patients using socioeconomic factors, and proactive care management programs have reduced preventable hospital admissions by 27% among high‐risk participants [8]. AI screening tools designed for resource‐limited settings include smartphone systems that detect lung disease with high accuracy, offering meaningful potential for health equity in underserved regions [9].

Deploying AI in clinical settings for low‐income populations raises substantial ethical and methodological concerns. Willingness to use AI tools varies by demographic and socioeconomic status, with lower income populations accepting these technologies less often, which complicates equitable implementation [10]. ML models also face fairness challenges because device types and socioeconomic access introduce bias into predictions [11]. Experts have identified eight challenges for equitable deployment in opioid use disorder treatment, including algorithmic transparency, clinical validation, and digital exclusion among low‐income individuals [12].

Ethical dimensions of AI and big data are structural features of health equity work. A scoping review of 243 articles found that these technologies reshape health determinants and policies [2]. Governance frameworks for digital healthcare integrate big data analytics with value‐based care principles to protect low‐income populations during health crises [13]. Responsible data use principles, including safety, transparency, fairness, and accountability, form a blueprint for interdisciplinary AI collaboration [14].

ML models incorporating socioeconomic variables consistently outperform traditional clinical markers in predicting risk. Deep learning models trained on behavioral and socioeconomic features show strong predictive performance for mortality outcomes [15]. During pandemics, ML methods integrating community mobility and census data outperform traditional models in forecasting disease spread [16]. Social determinants function as essential predictive signals rather than background context, and federated learning networks across hospital systems provide infrastructure for analyzing SDOH at scale [4].

Integrating AI and SDOH data advances value‐based healthcare. Governance frameworks identify big data analytics as essential for improving outcomes in resource‐constrained environments [13]. Studies in low‐income primary care settings confirm that household income and neighborhood position each independently relate to disease burden [17]. The Yale School of Public Health has called for big data infrastructure connecting SDOH to health outcomes while diversifying voices in AI development [1].

Significant gaps persist despite expanding literature. Few scoping reviews systematically characterize how AI and ML identify SDOH risks for low‐income individuals in United States health systems or examine interventions responding to identified risks. Existing research frequently separates methodology from SDOH content or overlooks economic vulnerability in safety‐net settings. The present review maps evidence on AI approaches used to identify SDOH and linked interventions in United States hospitals and clinical care settings.

## 2 Methods

### 2.1 Study Design

A scoping review was conducted following the Arksey and O'Malley methodological framework [18] and reported in accordance with the Preferred Reporting Items for Systematic Reviews and Meta‐Analyses Extension for Scoping Reviews (PRISMA‐ScR) [19]. The completed PRISMA‐ScR checklist is provided as Supporting Information for Review and Publication. Scoping review methodology was selected over systematic review methodology because the goal was to map the breadth of evidence on AI and ML applications for SDOH identification in low‐income clinical populations, not to synthesize effect estimates or address a single clinical question. Scoping reviews permit broader inclusion criteria, accommodate methodological heterogeneity, and are appropriate when a field is emerging and the boundaries of the literature remain undefined.

### 2.2 Research Question

Among low‐income individuals receiving care in health system settings, what AI and ML approaches have been used to identify SDOH risks, and what interventions, if any, have been implemented in response to those risks, across hospitals, integrated delivery networks, and other organized clinical care settings in the United States?

### 2.3 Registration

This scoping review was prospectively registered on the Open Science Framework (OSF) Registries before data extraction. The registration was submitted by the author on April 10, 2026, under an Open‐Ended Registration type. The registration is publicly accessible via the OSF Registries platform (Registration DOI: 10.17605/OSF. IO/GZ3Q8).

### 2.4 Eligibility Criteria

Studies were included if they: (1) applied or evaluated an AI or ML method (including but not limited to ML, deep learning, natural language processing, predictive modeling, neural networks, random forest, or related algorithmic methods); (2) addressed SDOH, social risk factors, or social needs as a primary or substantive focus; (3) included a population explicitly described as low‐income, economically disadvantaged, Medicaid‐enrolled, living in poverty, or characterized by socioeconomic deprivation; (4) were conducted within a hospital, integrated delivery network, or organized clinical care setting in the United States; and (5) were published as peer‐reviewed articles in English between January 2010 and January 2026.

For this review, a population was considered low income or economically disadvantaged when the original study explicitly identified the study population or a separately reported subgroup using at least one of the following criteria: individual or household income; income relative to a federal poverty measure; Medicaid enrollment or another means‐tested indicator of economic eligibility; an explicit description of the population as low income, living in poverty, or economically disadvantaged; or a study‐defined measure of socioeconomic or neighborhood deprivation. No single income threshold or uniform percentage of the federal poverty level was imposed because definitions and measures varied across studies. Medicaid enrollment and area‐level deprivation measures were treated as proxies for economic disadvantage rather than equivalent measures of individual household income. Race, ethnicity, clinical setting, and health status alone were not considered sufficient indicators of low‐income status.

Studies were excluded if they: lacked an applied AI or ML method (e.g., studies using only logistic regression, generalized estimating equations, or descriptive statistics); addressed SDOH only peripherally as background context; examined a general population without a low‐income subgroup analysis; were conducted in international settings, community‐only settings, public health agencies, or social service agencies outside a health system context; used SDOH variables solely as covariates to predict a clinical outcome without SDOH identification as a primary aim; or represented preprint versions superseded by a published study included in the review.

### 2.5 Search Strategy

All database searches were conducted through the EBSCO platform via the University of Massachusetts Lowell library institutional subscription. Four databases were searched: MEDLINE, CINAHL Plus with Full Text, PsycINFO, and Academic Search Ultimate. Searches were limited to peer‐reviewed publications from January 2010 through January 2026 to capture the period during which AI and ML applications in health system settings emerged as an active area of inquiry. The databases, search platform, search dates, applied limits, and complete search strategy are presented in Table 1.

**Table 1:** Database search strategy.

Databases	MEDLINE, CINAHL, PsycINFO, Academic Search Ultimate
Platform	EBSCO
Date searched	January 2026
Date range applied	January 2010—January 2026
Limiters	Peer‐reviewed publications; English language
Search string	(“artificial intelligence” OR “machine learning” OR “deep learning” OR “natural language processing” OR “predictive model*“ OR “neural network*“ OR “random forest” OR “algorithm*“) AND (“social determinants of health” OR “social determinant*“ OR “SDOH” OR “social risk*“ OR “social needs”) AND (“low income” OR “low‐income” OR “poverty” OR “Medicaid” OR “economically disadvantaged” OR “socioeconomic*“ OR “deprivation”) AND (“health system*“ OR “hospital*“ OR “integrated delivery” OR “electronic health record*“ OR “EHR” OR “clinical setting*“ OR “health care system*“)

The following search string was applied consistently across all four databases:

(“artificial intelligence” OR “machine learning” OR “deep learning” OR “natural language processing” OR “predictive model*“ OR “neural network*“ OR “random forest” OR “algorithm*“) AND (“social determinants of health” OR “social determinant*“ OR “SDOH” OR “social risk*“ OR “social needs”) AND (“low income” OR “low‐income” OR “poverty” OR “Medicaid” OR “economically disadvantaged” OR “socioeconomic*“ OR “deprivation”) AND (“health system*“ OR “hospital*“ OR “integrated delivery” OR “electronic health record*“ OR “EHR” OR “clinical setting*“ OR “health care system*“)

The search was restricted to four EBSCO databases to maximize reproducibility and transparency across a single standardized platform. This approach may have resulted in the omission of relevant literature indexed exclusively in other databases, including IEEE Xplore, ACM Digital Library, Scopus, and Embase, which contain substantial computer science and health informatics literature relevant to AI applications in health systems. Grey literature, conference proceedings, and non‐English language publications were outside the scope of this search. This scoping review prioritizes transparency and reproducibility; future searches should incorporate technical computing databases to ensure comprehensive coverage of the methodological literature.

### 2.6 Record Retrieval and Deduplication

Total records retrieved from each database were documented before deduplication. Table 2 summarizes retrieval counts. Duplicate records were removed using Rayyan before title and abstract screening.

**Table 2:** Search results and screening summary.

Database/category	Count
MEDLINE	172
CINAHL Plus with Full Text	50
APA PsycINFO	13
Academic Search Ultimate	74
Total records retrieved (pre‐deduplication)	309
Duplicates removed	100
Unique records for screening	209
Excluded at title/abstract screening	170
Proceeding to full‐text review	39
Excluded at full‐text review	22
INCLUDED for synthesis	17

### 2.7 Screening and Selection

All 209 unique records were screened at the title and abstract level by the primary reviewer and reviewed by two additional reviewers, both healthcare data and analytics professionals. The additional reviewers’ involvement was limited to reviewing screening decisions and was intended to reduce the potential for selection bias associated with a sole‐author review. Reviewers applied four prespecified exclusion criteria: (1) no AI or ML method applied; (2) no substantive SDOH focus; (3) population not explicitly identified as low‐income or Medicaid‐enrolled; and (4) setting not within a United States clinical health system. Inconsistent screening decisions were discussed among the three reviewers in an effort to reach consensus; when consensus could not be reached, the final decision was determined by majority vote. Because the process did not involve fully independent duplicate screening with separately retained reviewer‐level decisions, a chance‐corrected inter‐rater reliability statistic was not calculated.

**Figure 1:**
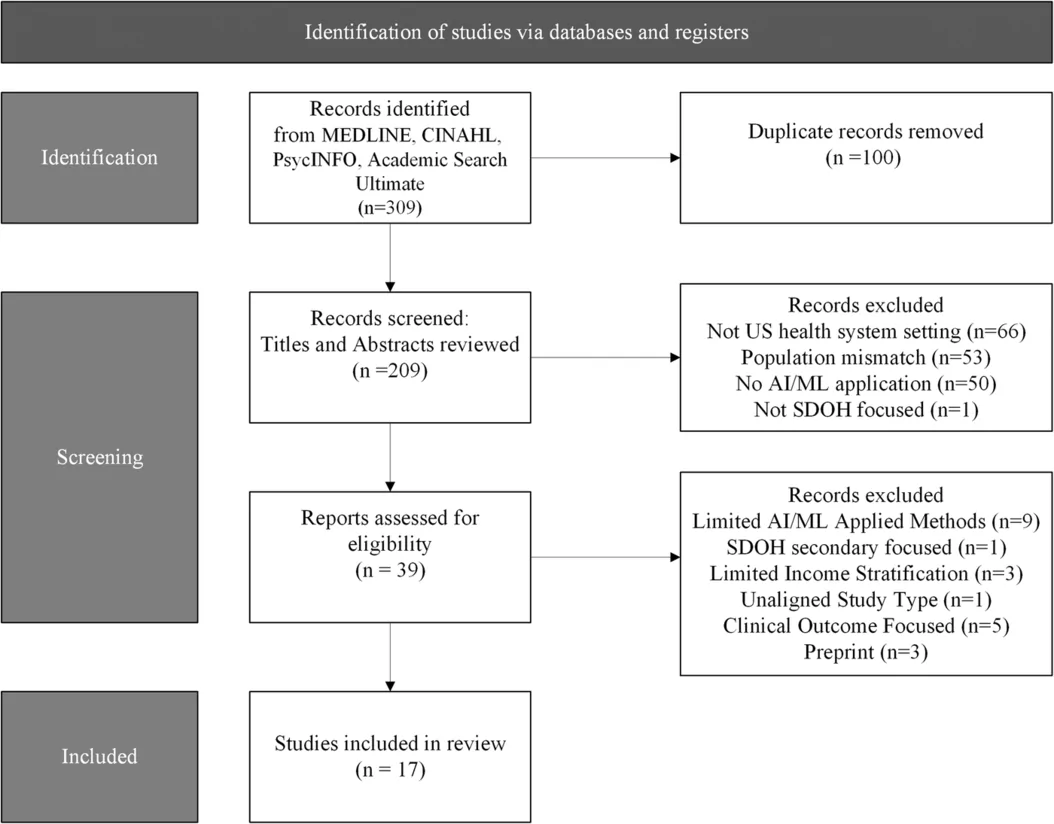
PRISMA‐ScR flow diagram.

Following title and abstract screening, 170 records were excluded. The primary reasons for exclusion were setting not within a United States clinical health system (*n* = 66), population not explicitly identified as low‐income or Medicaid‐enrolled (*n* = 53), no AI or ML application (*n* = 50), and no substantive SDOH focus (*n* = 1). Thirty‐nine records proceeded to full‐text review. After full‐text assessment, 22 additional studies were excluded: no AI or ML method applied (*n* = 9); SDOH used only as a covariate to predict a clinical outcome (*n* = 5); population not identified as low‐income or Medicaid‐enrolled (*n* = 3); duplicate publication or preprint superseded by an included published version (*n* = 3); SDOH not a primary focus (*n* = 1); and ineligible study type (*n* = 1). Seventeen studies met all eligibility criteria and were included in the final synthesis. The screening and selection process is presented in the PRISMA‐ScR flow diagram in Figure 1.

### 2.8 Data Extraction

Data were extracted from each included study using a standardized form capturing: author(s) and year, title, study design, population and clinical setting, AI or ML method applied, SDOH domains addressed, primary findings, and any interventions implemented in response to identified SDOH risks. Data extraction was completed by the primary reviewer using a standardized form. The two additional reviewers examined the completed extraction for completeness and consistency, and any questions identified during verification were resolved through discussion. The additional reviewers did not independently duplicate the complete data‐extraction process.

### 2.9 Quality Appraisal

Quality appraisal was conducted using the Mixed Methods Appraisal Tool (MMAT) and the Prediction Model Risk of Bias Assessment Tool (PROBAST). The MMAT assessed methodological quality using design‐specific criteria. PROBAST evaluated prediction‐model studies across four domains: participants, predictors, outcomes, and analysis. Signaling questions within each domain were used to judge risk of bias as low, high, or unclear. An overall low‐risk judgment required all applicable domains to be rated as low risk; a high‐risk judgment was assigned when at least one domain was rated as high risk; and an unclear judgment reflected insufficient information to determine risk. Applicability concerns were assessed separately by considering whether the study population, predictors, and outcomes aligned with the review question. For example, a study with appropriate participant selection and clearly defined predictors and outcomes could still receive a high overall risk‐of‐bias rating if the analysis lacked adequate validation or presented substantial potential for model overfitting. Consistent with scoping‐review methodology, appraisal results were used to contextualize the evidence and were not used as criteria for study exclusion.

## 3 Results

### 3.1 Overview of Included Studies

Seventeen studies met final inclusion criteria, published between 2018 and 2025. Study populations ranged from 1550 patients in a multi‐site cross‐sectional validation study to 14.2 million Medicaid recipients in a national predictive modeling study. Settings included academic medical centers, integrated delivery networks, safety‐net hospitals, Veterans Health Administration facilities, and multi‐state Medicaid administrative data environments. All 17 studies used quantitative methods: 10 employed predictive ML models, five applied NLP, and two used unsupervised algorithmic clustering. EHR data were used in 16 of 17 studies, often linked with Medicaid claims, census or American Community Survey neighborhood data, or cross‐sector administrative records from human services and criminal justice systems.

### 3.2 Study Summary Table

Table 3 summarizes each included study by author and year, title, study design, population and setting, and key findings.

**Table 3:** Summary of included studies (*n* = 17).

Author (Year)	Title	Study design	Population/setting	Key findings
Hall et al. (2025) [20]	Unsupervised Learning Using EHR and Census Data to Identify Hypertension Subphenotypes	Retrospective cohort; hierarchical clustering (unsupervised ML)	40,686 adults; Medicaid/EHR; Florida 2012–2021	Five hypertension subphenotypes identified; one predominantly younger Black women with higher poverty and Medicaid enrollment; social factors integral to hypertension heterogeneity
Huang et al. (2024) [21]	A fair Individualized Polysocial Risk Score for Identifying Increased Social Risk in Type 2 Diabetes	Retrospective cohort; ML predictive modeling with fairness optimization	10,192 T2D patients; UF Health system	C‐statistic 0.71; top 5% risk score had 28.1% hospitalization rate (~13x bottom decile); fairness optimization reduced bias without major accuracy loss
Patel et al. (2025) [22]	Predicting Quality Measure Completion Among 14 Million Low‐Income Patients Enrolled in Medicaid	Retrospective cohort; ML predictive modeling	14.2 million Medicaid recipients	AUROC 0.88, F1 0.69; ML outperformed non‐predictive outreach by 32.5 percentage points; SDOH data further improved performance
Juhn et al. (2022) [23]	Assessing Socioeconomic Bias in ML Algorithms: Case Study of the HOUSES Index	Retrospective cohort; bias assessment of existing ML models	Pediatric asthma cohort; single site	Balanced error rate ratio 1.35 for lowest versus other SES quartiles; 41% of lower‐SES children had missing EHR records versus 24% of higher‐SES peers; EHR data gaps drive algorithmic bias
Gupta et al. (2025) [24]	Associations of Longitudinal BMI‐Percentile Classification Patterns with Neighborhood‐Level SDOH	Retrospective longitudinal cohort; algorithmic clustering	36,910 children aged 0–7; five East Coast states 2012–2019	Five BMI trajectories identified; children in higher‐risk groups significantly more likely in high‐poverty, high‐unemployment neighborhoods; neighborhood SDOH shapes childhood obesity trajectories
Grant et al. (2024) [25]	Predicting Self‐Reported Social Risk in Medically Complex Adults Using EHR Data	Retrospective cohort; ML with prospective external validation	Development cohort of 11,999 adults with two or more chronic conditions; external validation cohort of 2,820 non‐Medicaid members; Kaiser Permanente Northern California	EHR‐based model predicted food insecurity, financial distress, or housing instability with an AUC of 0.68; performance remained robust across racial and ethnic strata; external validation was conducted in a separate cohort.
Choi et al. (2025) [26]	Predicting ED Visits for Non‐Traumatic Dental Conditions Among Medicaid Beneficiaries	Retrospective cohort; ML ensemble models	6.4 million Medicaid beneficiaries aged 19–64; four states	C‐statistics 0.70–0.73; accuracy 0.90–0.95; specificity 0.93–0.97; ML consistently outperformed logistic regression; sociodemographic risk factors identified
Gregory et al. (2025) [27]	Development and Validation of Computable Social Phenotypes for Health‐Related Social Needs	Cross‐sectional validation; rule‐based and ML (XGBoost)	1550 patients; three health systems; two states	Both approaches showed poor‐to‐fair performance versus patient‐reported reference standards; accuracy lower for non‐White and Hispanic patients; EHR structured data insufficient for reliable HRSN identification
Goldstein et al. (2024) [28]	Poverty and Suicidal Ideation Among Hispanic Mental Health Care Patients Leading up to the COVID‐19 Pandemic	Retrospective observational cohort; NLP extraction from clinical notes	22,968 Hispanic patients (52,703 patient‐year observations); 23 facilities 2010–2020	NLP extracted poverty‐related SDOH and suicidal ideation from unstructured notes; poverty significantly associated with higher odds of suicidal ideation pre‐pandemic
Yao et al. (2023) [29]	Automated Identification of Eviction Status from EHR Notes	Retrospective NLP model development and validation	5,000 VHA EHR notes	KIRESH‐Prompt achieved MCC 0.747 for eviction period prediction; outperformed BioBERT and BioClinicalBERT; planned deployment as VHA eviction surveillance system
Holcomb et al. (2022) [30]	Predicting Health‐Related Social Needs in Medicaid and Medicare Populations Using Machine Learning	Retrospective cohort; ML predictive modeling	Medicare and Medicaid beneficiaries; CMS Accountable Health Communities Model	AUROC 0.59–0.68; composite any‐HRSN achieved AUROC 0.68; all models outperformed baseline assumption that Medicaid coverage alone predicted social needs
Rigdon et al. (2024) [31]	Predicting Food Insecurity in a Pediatric Population Using the EHR	Retrospective longitudinal cohort; ML predictive modeling	Pediatric primary care; academic clinic 2017–2021	Census block‐level data with 12‐month lookback yielded best performance; among first pediatric studies using ML to predict food insecurity; further refinement needed for populations with infrequent care
Lo‐Ciganic et al. (2021) [32]	Integrating Human Services and Criminal Justice Data with Claims Data to Predict Opioid Overdose Among Medicaid Beneficiaries	Retrospective prognostic cohort; gradient boosting ML; linked administrative datasets	237,259 Medicaid beneficiaries; Allegheny County, PA 2015–2018; 290 predictors	Cross‐domain data linkage substantially improved overdose risk prediction versus claims alone; incarceration history and social service utilization captured key SDOH signals
Derton et al. (2023) [33]	NLP Methods to Empirically Explore Social Contexts and Needs in Cancer Patient Notes	Retrospective NLP analysis; log odds ratios; variational autoencoder topic model	230,325 clinical notes; 5,285 patients; academic cancer center 2007–2019	Non‐White and low‐income insurance patients disproportionately associated with housing and transportation terms; White/privately insured patients referenced physical activity; clinical notes contain detectable social context signals
Kasthurirathne et al. (2018) [34]	Assessing the Capacity of SDOH Data to Augment Predictive Models Identifying Patients in Need of Wraparound Social Services	Retrospective cohort; random forest ML	Safety‐net hospital; community‐linked SDOH data	Random forest predicted social service referral needs with considerable accuracy; community‐level SDOH added meaningful predictive value beyond clinical variables alone; among earliest demonstrations of SDOH‐augmented prediction in clinical settings
Sato et al. (2025) [35]	NLP for Identification of Hospitalized People Who Use Drugs	Retrospective cohort; NLP for case identification	4,548 hospitalizations; Tufts Medical Center 2020–2022	NLP positive predictive value 54%; outperformed biomarkers, prescriptions, and diagnosis codes alone; minoritized and lower SVI patients systematically missed by traditional methods; NLP improves identification equity
Philipp et al. (2025) [36]	Impact of a Proactive Patient Assistance Program for Diverse Ambulatory Oncology Patients	Retrospective cohort; proactive financial assistance program evaluation	Ambulatory oncology infusion patients; NCI‐designated cancer center; post‐July 2022	10% Black, 39% Hispanic patients; 95% in high‐SVI counties; proactive data‐driven program reached patients at greatest HRSN and financial distress risk; supports equitable cancer care

### 3.3 AI and ML Approaches

Predictive ML modeling was the most common approach, used in 10 studies [21, 22, 23, 25, 26, 27, 30, 31, 32, 34]. Methods included gradient boosting [32], random forest [26, 34], ensemble models [26], XGBoost [27], and logistic regression‐based ML pipelines with fairness optimization [21]. NLP was applied in five studies to extract SDOH information from unstructured clinical notes [28, 29, 33, 35] and to compare NLP performance against structured EHR data [27]. Unsupervised clustering, including hierarchical clustering [20] and trajectory‐based algorithmic grouping [24], identified patient subphenotypes linked to SDOH exposure.

### 3.4 SDOH Domains Addressed

Financial hardship and poverty (*n* = 9) were the most frequently addressed SDOH domains, followed by housing instability and eviction (*n* = 7), food insecurity (*n* = 5), and social service or care management needs (*n* = 5). Neighborhood‐level socioeconomic deprivation, indexed through census or Area Deprivation Index variables, appeared in 10 studies. Substance use, including opioid overdose risk and drug use identification, was the primary focus in two studies. Transportation barriers, social isolation, and employment instability were addressed in a smaller subset.

### 3.5 Predictive Performance

Among the 10 predictive ML studies, AUROC ranged from 0.59 to 0.88. Patel et al. [22] reported the highest performance, with an AUROC of 0.88 and F1 score of 0.69 across nine quality measure gaps in 14.2 million Medicaid recipients, outperforming non‐predictive outreach strategies by 32.5 percentage points. Holcomb et al. [30] reported the lowest AUROC range (0.59–0.68), with the composite any‐HRSN outcome achieving the highest performance within that study. Huang et al. [21] reported a C‐statistic of 0.71 with fairness‐optimized social risk scoring in type 2 diabetes, and Choi et al. [26] reported C‐statistics of 0.70–0.73 for Medicaid dental emergency department visit prediction. NLP models demonstrated strong performance: Yao et al. [29] reported a Matthews Correlation Coefficient of 0.747 for eviction status prediction, and Sato et al. [35] achieved a positive predictive value of 54% for identifying hospitalized people who use drugs, surpassing identification by diagnostic codes, biomarkers, or prescriptions alone.

### 3.6 Algorithmic Fairness and Bias

Several studies documented differential model performance across socioeconomic and racial subgroups. Juhn et al. [23] found that asthma prediction models performed significantly worse for children in the lowest socioeconomic status quartile, with a balanced error rate ratio of 1.35 compared with higher‐SES peers, attributable in part to greater EHR data incompleteness in lower‐SES patients (41% missing relevant records vs. 24% in higher‐SES children). Gregory et al. [27] found that computable social phenotypes derived from structured EHR data performed poorly for non‐White and Hispanic patients relative to White patients, even using XGBoost with 78 features. Huang et al. [21] applied fairness optimization using SHAP‐based explanations and algorithmic debiasing, showing that fairness constraints can reduce disparate performance without substantially compromising overall predictive accuracy. Grant et al. [25] found no meaningful differential performance across racial and ethnic subgroups in their EHR‐based social risk model, one of the few studies reporting equity metrics with favorable results.

### 3.7 Data Sources and Integration

Studies varied substantially in data source complexity. Most relied on EHR data alone or linked EHR with census or administrative neighborhood data. Lo‐Ciganic et al. [32] achieved the most complex data integration, linking Medicaid claims with human services records and criminal justice data to predict opioid overdose risk, and showed that cross‐sector data linkage substantially improves predictive performance over claims data alone. Kasthurirathne et al. [34] augmented clinical EHR data with community‐level socioeconomic and public health datasets, one of the earliest demonstrations that SDOH‐enriched models improve social service referral prediction. Rigdon et al. [31] supplemented EHR data with American Community Survey neighborhood data at the census block level. Yao et al. [29] and Sato et al. [35] relied solely on clinical note text, underscoring the information density in unstructured documentation.

### 3.8 Interventions in Response to Identified Risks

Most included studies focused on risk identification rather than intervention. Philipp et al. [36] was the only study to evaluate a structured response to identified SDOH risk, describing a proactive patient financial assistance program at a National Cancer Institute‐designated comprehensive cancer center that used data‐driven screening to identify ambulatory oncology patients at risk of financial toxicity and unmet HRSN. The program served a predominantly minority and low‐income population, with 95% of participants residing in high Social Vulnerability Index counties. Patel et al. [22] showed that ML‐guided outreach could improve quality gap closure efficiency in Medicaid populations, though the study evaluated outreach improvement rather than a clinical intervention. Cohen Marill (2025), cited in the introduction, described AI‐paired care management programs reducing preventable admissions, but this report was not included as a primary study. No included study reported a randomized or quasi‐experimental evaluation of an SDOH‐responsive clinical or community intervention triggered by ML or AI identification.

### 3.9 Quality Appraisal

Table 4 presents PROBAST risk‐of‐bias ratings, and Table 5 presents MMAT quality ratings for all 17 included studies.

**Table 4:** PROBAST risk of bias assessment (*n* = 17).

Author (Year)	D1: Participants	D2: Predictors	D3: Outcome	D4: Analysis	Overall ROB	Applicability	Key Concerns
Hall et al. (2025) [20]	Low	Low	High	High	High	Low concern	High ROB: unsupervised outcome lacks gold standard; no external validation
Huang et al. (2024) [21]	Low	Low	Low	High	High	Low concern	High ROB: no external validation; calibration not reported; single health system
Patel et al. (2025) [22]	Low	Low	Low	Low	Low	Low concern	Low ROB: large national Medicaid sample; well‐defined outcomes; external validation; robust ML methodology
Juhn et al. (2022) [23]	Low	Low	Unclear	High	High	Unclear	High ROB: no gold standard for SES outcome; single site; no calibration
Gupta et al. (2025) [24]	Low	Low	High	High	High	Low concern	High ROB: unsupervised trajectory outcome; no external validation; class solution subjective
Grant et al. (2024) [25]	Low	Low	Low	High	High	Low concern	High ROB: small sample; overfitting risk with complex ML
Choi et al. (2025) [26]	Low	Low	Low	High	High	Low concern	High ROB: no external validation; single‐state Medicaid claims; calibration not fully reported
Gregory et al. (2025) [27]	Low	Low	Low	Low	Low	Low concern	Low ROB: multi‐site validation; chart review gold standard; appropriate NLP methods; full performance metrics
Goldstein et al. (2024) [28]	Low	Low	Unclear	High	High	Unclear	High ROB: EHR documentation bias for suicidal ideation; single site; no external validation
Yao et al. (2023) [29]	Low	Low	Low	High	High	Low concern	High ROB: internal validation only; single health system; limited generalizability
Holcomb et al. (2022) [30]	Low	Low	Low	High	High	Low concern	High ROB: modest AUROC (0.59–0.68); internal validation only; calibration unreported
Rigdon et al. (2024) [31]	Low	Low	Low	High	High	Low concern	High ROB: single‐site; internal validation only; limited sample size
Lo‐Ciganic et al. (2021) [32]	Low	Low	Low	High	High	Low concern	High ROB: single‐state Medicaid; internal validation only; state‐specific data linkage
Derton et al. (2023) [33]	Low	Low	Low	High	High	Low concern	High ROB: single‐site NLP; internal validation only; note style limits applicability
Kasthurirathne et al. (2018) [34]	Low	Low	Unclear	High	High	Unclear	High ROB: referral as imperfect proxy; single site; internal validation only
Sato et al. (2025) [35]	Low	Low	Low	High	High	Low concern	High ROB: single‐site NLP; internal validation only; no external multi‐site validation
Philipp et al. (2025) [36]	Low	Unclear	Unclear	High	High	Unclear	High ROB: limited methodological transparency; unclear outcome definition; no external validation

**Table 5:** Mixed methods appraisal tool (MMAT) quality ratings (*n* = 17).

Author (Year)	MMAT Design	S1: Clear RQ?	S2: Data fits RQ?	Q1: Representativeness	Q2: Measurement	Q3: Complete Data	Q4: Confounders	Q5: Follow‐up	Overall Quality
Hall et al. (2025) [20]	Quant. Descriptive	Y	Y	Y	Y	Y	N/A	Y	High
Huang et al. (2024) [21]	Quant. Non‐Randomized	Y	Y	Y	Y	Y	Y	Y	High
Patel et al. (2025) [22]	Quant. Non‐Randomized	Y	Y	Y	Y	Y	Y	Y	High
Juhn et al. (2022) [23]	Quant. Non‐Randomized	Y	Y	?	Y	?	Y	Y	Moderate‐High
Gupta et al. (2025) [24]	Quant. Non‐Randomized	Y	Y	Y	Y	Y	Y	Y	High
Grant et al. (2024) [25]	Quant. Non‐Randomized	Y	Y	Y	Y	Y	?	Y	Moderate‐High
Choi et al. (2025) [26]	Quant. Non‐Randomized	Y	Y	Y	Y	Y	?	Y	Moderate‐High
Gregory et al. (2025) [27]	Quant. Descriptive	Y	Y	Y	Y	Y	N/A	Y	High
Goldstein et al. (2024) [28]	Quant. Non‐Randomized	Y	Y	Y	Y	?	?	Y	Moderate
Yao et al. (2023) [29]	Quant. Descriptive	Y	Y	Y	Y	Y	N/A	Y	High
Holcomb et al. (2022) [30]	Quant. Non‐Randomized	Y	Y	Y	Y	Y	?	Y	Moderate‐High
Rigdon et al. (2024) [31]	Quant. Non‐Randomized	Y	Y	Y	Y	Y	?	Y	Moderate‐High
Lo‐Ciganic et al. (2021) [32]	Quant. Non‐Randomized	Y	Y	Y	Y	Y	Y	Y	High
Derton et al. (2023) [33]	Quant. Descriptive	Y	Y	Y	Y	Y	N/A	Y	High
Kasthurirathne et al. (2018) [34]	Quant. Non‐Randomized	Y	Y	Y	Y	Y	?	Y	Moderate‐High
Sato et al. (2025) [35]	Quant. Descriptive	Y	Y	Y	Y	Y	N/A	Y	High
Philipp et al. (2025) [36]	Quant. Non‐Randomized	Y	Y	Y	?	?	N	?	Moderate

Two studies achieved low overall PROBAST risk of bias: Patel et al. [22] and Gregory et al. [27]. The remaining 15 studies were rated as high overall risk of bias, driven predominantly by Domain 4 (Analysis), reflecting the near‐universal absence of external validation. Domain 1 (Participants) and Domain 2 (Predictors) received low bias ratings in 17 and 16 studies, respectively, indicating sound participant selection and predictor ascertainment. Domain 3 (Outcome) received unclear or high bias ratings in six studies, primarily those using unsupervised clustering outcomes or social service referral as a proxy for social need.

MMAT quality ratings classified nine studies as high quality, six as moderate‐high, and two as moderate. No study was rated as low quality. Moderate‐rated studies reflected limited confounder adjustment, unclear outcome definitions, or reduced methodological transparency. High‐quality ratings corresponded to studies with well‐defined research questions, appropriate ML methods, complete data handling, and multi‐site or externally validated designs.

## 4 Discussion

Seventeen studies published between 2018 and 2025 applied AI or ML methods to identify SDOH risks in low‐income populations within United States health system settings. ML‐based predictive modeling and NLP emerged as the dominant methodological approaches, with EHR data serving as the primary data source in nearly all studies. Studies demonstrated meaningful predictive performance across SDOH domains including food insecurity, housing instability, poverty, and opioid overdose risk. Critical gaps persist despite this progress: external validation was uncommon, only two studies achieved low PROBAST overall risk of bias, algorithmic disparities affecting low‐income and racial minority subgroups were documented repeatedly, and translation of ML‐identified SDOH risk into structured clinical or social interventions remained largely underdeveloped.

Consistent reliance on EHR data reflects the infrastructure available to health systems and the operational feasibility of integrating clinical records with predictive models. Studies linking EHR data with census‐derived neighborhood indicators, Medicaid claims, or cross‐sector administrative data achieved stronger predictive performance and captured SDOH dimensions that clinical documentation alone misses. Lo‐Ciganic et al. [32] showed that integrating human services and criminal justice data with Medicaid claims substantially improved opioid overdose risk prediction. Kasthurirathne et al. [34] showed that community‐level SDOH data added predictive value for social service referral need beyond clinical variables alone. Health systems willing to invest in data partnerships with public sector and social service agencies can substantially improve SDOH identification capacity.

NLP emerged as a distinct and productive pathway for SDOH identification. Unstructured clinical notes contain detailed social histories, patient‐reported circumstances, and provider observations that structured EHR fields fail to capture. Yao et al. [29] developed an eviction detection model that outperformed state‐of‐the‐art biomedical language models. Derton et al. [33] showed that notes from non‐White and low‐income patients were linguistically distinct from those of White and privately insured patients, with housing and transportation terms appearing at higher rates in the former group. Sato et al. [35] found that NLP identified people who use drugs who were systematically missed by diagnostic codes alone, with minoritized and lower vulnerability‐index patients more likely to appear only in the NLP‐identified subgroup. NLP offers both identification capacity and a lens for documenting disparities that structured data cannot reveal.

Algorithmic bias against low‐income and racial minority populations was a recurring finding and represents one of the most consequential challenges in the field. Juhn et al. [23] attributed worse ML performance for lower SES children directly to greater EHR data incompleteness: when training data systematically underrepresent the social circumstances of lower income patients, models underperform for the populations they are intended to serve. Gregory et al. [27] reinforced this concern, finding that computable social phenotypes performed poorly for non‐White and Hispanic patients even with complex ML methods. Huang et al. [21] showed that fairness‐constrained model optimization can reduce disparate performance, and Grant et al. [25] reported equitable cross‐group performance in their social risk model, indicating that algorithmic equity is achievable with deliberate methodological attention. Health systems deploying SDOH prediction models should conduct prospective fairness audits stratified by race, ethnicity, and income level before clinical implementation.

A notable gap in this literature is the near‐complete absence of intervention evaluation. Philipp et al. [36] was the only study to describe a structured programmatic response to SDOH risk identified through data‐driven methods. ML‐based risk identification creates the technical precondition for targeted intervention, but identification alone does not reduce social need. The gap between prediction and action mirrors broader challenges in precision medicine and population health management, where the capacity to identify who is at risk outpaces the ability to deliver effective responses. Future research must prioritize rigorous evaluation of interventions that use ML‐identified SDOH risk as the trigger for care navigation, community resource linkage, or social service referral, using randomized or quasi‐experimental designs where feasible.

Single‐site studies with internal validation only represent a structural limitation of this evidence base. Fifteen of 17 studies were rated as high overall PROBAST risk of bias largely because external validation was absent, limiting confidence in the generalizability of reported performance metrics. ML models trained on EHR data from a single health system reflect the documentation practices, patient mix, and operational context of that institution, and performance frequently degrades when models are applied to new settings. The two studies achieving low PROBAST risk of bias, Patel et al. [22] and Gregory et al. [27], both incorporated multi‐site or national data and applied external validation procedures. Multi‐site consortium approaches and pre‐registered external validation studies should become standard expectations for the field.

Study concentration in certain SDOH domains reflects both clinical relevance and data availability. Financial hardship, housing instability, and food insecurity are the most frequently screened SDOH in health system settings, corresponding to domains most commonly captured in structured EHR screening tools such as PRAPARE and AHC‐HRSN. Employment, childcare, domestic violence, and civic participation remain underrepresented in the AI and ML literature, likely because they are documented less consistently in clinical records. Expanding SDOH capture in both structured screening and unstructured clinical documentation would broaden the domain coverage achievable through AI‐based identification.

## 5 Limitations

Several limitations apply to this review. The search was restricted to four databases available through the EBSCO platform and did not include IEEE Xplore, ACM Digital Library, Scopus, or Embase, which index substantial computer science and health informatics literature. Grey literature, conference proceedings, and non‐English publications were excluded. Relevant studies may have been missed as a result. The inclusion criteria required explicit identification of low‐income, Medicaid‐enrolled, or economically disadvantaged populations, which may have excluded studies that applied AI to general clinical populations with unpublished low‐income subgroup analyses. Because this is a scoping review, no formal quantitative synthesis or meta‐analysis was conducted, and the heterogeneity of methods, populations, and outcomes across included studies precludes pooled effect estimation. Quality appraisal was not used as an exclusion criterion, consistent with scoping review methodology, meaning methodologically weaker studies were included alongside stronger ones. Finally, the rapid pace of AI development in healthcare means that studies published after January 2026 are not captured in this review.

## 6 Conclusion

AI and ML approaches show meaningful capacity to identify SDOH risks among low‐income populations within United States health systems, with predictive ML modeling and NLP providing the strongest and most consistent evidence. EHR data, enriched with census‐linked neighborhood indicators or cross‐sector administrative records, supports effective SDOH identification across multiple domains. Algorithmic bias against low‐income and racial minority subgroups is a documented and consequential problem that requires equity‐centered model development and prospective fairness auditing. External validation, intervention evaluation, and programmatic implementation remain substantially underdeveloped. Addressing these gaps requires investment in multi‐site data infrastructure, rigorous validation protocols, and research designs that connect ML‐identified SDOH risk to measurable improvements in care and social outcomes for the populations most in need.

## Author Contributions

**Anthony Vincent Razzano:** conceptualization, methodology, data curation, formal analysis, writing – original draft, writing – review and editing.

## Funding

The author has nothing to report.

## Ethics Statement

Ethical approval was not required for this scoping review because it involved the synthesis of previously published information and did not involve direct participation by human participants or access to identifiable private information. Informed consent was therefore not applicable.

## Conflicts of Interest

The author declares no conflicts of interest.

## Author Declaration

The sole author has read and approved the final version of the manuscript. Anthony Vincent Razzano, the corresponding author and manuscript guarantor, had full access to all of the data in this study and takes complete responsibility for the integrity of the data and the accuracy of the data analysis.

## Data Synthesis Statement

Owing to heterogeneity in study populations, AI and machine learning methods, SDOH domains, outcome definitions, and reported performance measures, findings were synthesized descriptively. No meta‐analysis, pooled effect estimation, or original statistical hypothesis testing was performed. Statistical measures reported in this review, including discrimination and classification‐performance metrics, were extracted from the included studies and are presented with sufficient context to identify the measure and comparison being reported.

## Transparency Statement

Anthony Vincent Razzano, the manuscript guarantor, affirms that this manuscript is an honest, accurate, and transparent account of the study being reported; that no important aspects of the study have been omitted; and that any discrepancies from the study as planned and registered have been explained.

## Data Availability

Data sharing not applicable to this article as no datasets were generated or analysed during the current study. Data sharing is not applicable to this article, as no datasets were generated or analyzed beyond the published studies cited herein. All included studies are cited in the reference list and are publicly available through their respective sources.
